# Prevalence of chronic renal failure stage 3 or more in HIV-infected patients in Antwerp: an observational study

**DOI:** 10.1186/1758-2652-13-S4-P89

**Published:** 2010-11-08

**Authors:** AW Colson, E Florence, H Augustijn, GA Verpooten, L Lynen, E Gheuens

**Affiliations:** 1 University Hospital Antwerpen, Nephrology-Hypertension, Edegem, Belgium; 2 Institute of Tropical Medicine Antwerp, Clinical Sciences, Antwerp, Belgium; 3 Nederlandstalige Belgische Vereniging voor Nefrologie, Edegem, Belgium; 4 ZNA campus Stuivenberg, Nephrology, Antwerp, Belgium

## 

The introduction of Highly Active Antiretroviral Therapy has transformed HIV-infection from an inevitably lethal disease to a chronic condition with a life expectancy comparable to that of people with diabetes mellitus.

In recent years it has become evident that people living with HIV/AIDS have an increased risk of developing cardiovascular disease and it is expected that the prevalence of chronic kidney disease will rise accordingly. To investigate the prevalence of chronic kidney disease in patients with HIV we conducted a retrospective observational analysis using the clinical database of a large centre (Institute of Tropical Medicine) in the urban area of Antwerp, Belgium. The prevalence of chronic kidney disease among HIV infected subjects was found to be 3.0%. The development of chronic kidney disease was associated with age above 50 years, lower CD4 cell counts and Caucasian origin. Screening for chronic renal disorders and prevention of evolution toward chronic renal failure is a crucial challenge in the management of people living with HIV/AIDS.

